# Relationship between Sport-Related Concussion and Sleep Based on Self-Report and Commercial Actigraph Measurement

**DOI:** 10.1089/neur.2021.0008

**Published:** 2021-04-26

**Authors:** Ciaran M. Considine, Daniel L. Huber, Anna Niemuth, Danny Thomas, Michael A. McCrea, Lindsay D. Nelson

**Affiliations:** ^1^ Department of Neurology, Vanderbilt University Medical Center, Nashville, Tennessee, USA.; ^2^ Department of Neurosurgery, Medical College of Wisconsin, Milwaukee Wisconsin, USA.; ^3^ Department of Pediatrics, Medical College of Wisconsin, Milwaukee Wisconsin, USA.; ^4^ Department of Neurology, Medical College of Wisconsin, Milwaukee Wisconsin, USA.

**Keywords:** actigraphy, football, mHealth mobile health technology, sleep, sport-related concussion

## Abstract

Sleep-wake disturbance (SWD) results from sport-related concussion (SRC) and may increase risk of protracted post-injury symptoms. However, methodological limitations in the extant literature limit our understanding of the role of SWD in SRC. This study examined the association between acute/subacute SRC and two sleep behaviors—sleep duration and efficiency—as measured by self-report and commercially available actigraphy (CA) in a sample of football players enrolled in a larger prospective longitudinal study of concussion. Fifty-seven high school and Division 3 male football players with SRC (mean [M] age = 18.00 years, standard deviation [SD] = 1.44) and 26 male teammate controls (M age = 18.54 years, SD = 2.21) were enrolled in this prospective pilot study. Sleep duration and sleep efficiency were recorded nightly for 2 weeks (starting 24–48 h post-injury in the SRC group) via CA and survey delivered via mobile application. There was no significant relationship between SRC and objectively recorded sleep measures, a null finding. However, the SRC group reported a brief (3-day) reduction in sleep efficiency after injury (M SRC = 82.18, SD = 12.24; M control = 89.2, SD = 4.25; *p* = 0.013; Cohen's *d* = 0.77), with no change in sleep duration. Self-reported and actigraph-assessed hours of sleep were weakly and insignificantly correlated in the SRC group (*r* = −0.21, *p* = 0.145), whereas they were robustly correlated in the non-injured control group (*r* = 0.65, *p* = 0.004). SWD post-SRC was not observed in objectively measured sleep duration or sleep efficiency and was modest and time-limited based on self-reported sleep efficiency. The weak correlation between self-reported and objective sleep behavior measures implies that subjective experience of SWD post-SRC may be due to factors other than actual changes in these observable sleep behaviors. Clinically, SWD in the early-subacute stages of recovery from SRC may not be adequately measurable via current CA. Subjective SWD may require alternative methods of evaluation (e.g., clinical actigraph or sleep study).

## Introduction

Mild traumatic brain injury (mTBI) accounts for the vast majority of medically treated TBIs (85+%).^1,2^ Sleep-wake disturbance (SWD) is a frequent complication of mTBI, and one population-based longitudinal study identified a rise from 10% incidence pre-injury to a 2-week post-injury incidence of 65% and 1-year post-injury incidence of 41%. Sport-related concussion (SRC), a subset of mTBI, is also associated with a significant incidence of SWD symptoms, although it is reported to be less prominent than in non-sport mTBI.^3^

The presence of SWD is a significant risk factor of protracted concussion-related symptomatology following both sport- and non-sport-related concussion (associated with a three- to four-fold increase in time to recovery).^3^ “Synaptic activity” refers to the continuous and plastic potentiation and depression of synaptic associations, which incorporates a series of complex cellular and molecular processes that includes receptor delivery and phosphorylation. This activity, which consumes 80% of the brain's energy during the wakeful period, returns to baseline levels during sleep.^4^ Given the neurometabolic crisis and increased energy demands that follow a concussion, restorative processes such as sleep may be instrumental in facilitating brain recovery.^5^ Ultimately, sleep disturbance may cause or intensify a variety of concussion-related comorbidities such as mood problems, fatigue, cognitive deficits, pain, and functional impairments that ultimately compromise recovery.^6,7^ Current literature suggests that SWD is a common sequelae of concussion but, once present, may independently contribute to other symptoms common to concussion (e.g., pain, depression, fatigue, cognitive dysfunction), thereby acting as a perpetuating factor.^3,8^

Although the broad incidence of SWD symptoms following mTBI and SRC are well documented, the acute-to-subacute characteristics of sleep-wake behavioral and physiological changes are less clear, in large part due to the difficulty of accurately measuring sleep-wake functions outside of the inpatient setting. A recent working group on mTBI and sleep identified research questions and actionable recommendations to guide sleep mTBI research methodology.^9^ The group noted that prospective longitudinal human studies are needed to replicate the fluctuating characteristics of post-injury SWD described in animal models. Additionally, the working group pointed to the need to characterize how different measures of SWD relate to one another following mTBI.

Self-reported sleep metrics have limited validity for estimating sleep behaviors or physiology due to reliable but weak associations between the subjective and objective methods. This poses a problem when circadian sleep-wake dysfunction is of interest, as multi-night polysomnography is intrusive and resource-heavy. Actigraphy uses a small device attached to an individual's body to continuously monitor movement (and sometimes light-exposure), which can then be used to estimate sleep-wake and circadian rhythm parameters via specialized algorithms. The method provides an objective metric of behavioral activity that correlates strongly with polysomnography-based measures of sleep-wake state—an attractive option due to the relative ease of using actigraphy clinically.^10^

With the aim of better understanding the prevalence, nature, and course of SWD after SRC, the current pilot prospective study collected both self-report and commercial actigraph sleep measurements in a sample of high school and collegiate football players who were enrolled in a larger prospective longitudinal study of SRC. Participants were enrolled in either the acute post-concussion period (<48 h) or after identification as a healthy control, and they then provided sleep data for 2 weeks (subacute period for SRC participants). The main objectives of the study were: 1) to characterize the relationship between subjective and commercial actigraphy based measures of sleep-wake function and subacute SRC, and 2) to estimate the relationship between objective and concurrent self-reported sleep-wake functioning measurements to inform clinicians and researchers interested in characterizing sleep in the SRC population.

## Methods

### Subjects

Participants in this sleep-monitoring study were recruited from a large prospective study of SRC that enrolled athletes from nine high schools and four Division 3 colleges around the greater Milwaukee, Wisconsin, region between the 2015 and 2017 football seasons. For this sub-study, athletes monitored after concussion or who served as healthy teammate controls for concussed participants during the 2016 and 2017 seasons were eligible to participate. A total of 58 concussed and 26 control athletes were enrolled in the sub-study. (One individual served as a control and, later, a concussed case.) Controls were selected to match the concussed athletes based on school, sports team, pre-season Wechsler Test of Adult Reading (WTAR) performance, self-reported cumulative grade point average, and age. All study procedures were approved by the Medical College of Wisconsin's Institutional Review Board. Adult participants or parents of minor participants provided written informed consent, whereas minor participants provided written assent. One concussed subject provided no valid sleep data, yielding 57 concussed and 26 control cases for analysis.

The definition of concussion used in this study is based on that of the study sponsor, the United States Department of Defense: “mTBI is defined as an injury to the brain resulting from an external force and/or acceleration/deceleration mechanism from an event such as a blast, fall, direct impact, or motor vehicle accident which causes an alteration in mental status typically resulting in the temporally-related onset of symptoms such as headache, nausea, vomiting, dizziness/balance problems, fatigue, insomnia/sleep disturbances, drowsiness, sensitivity to light/noise, blurred vision, difficulty remembering, and/or difficulty concentrating.”^11^ This sponsor-based definition parallels that of the 2017 Concussion in Sport Group's definition with regard to causal factors, acute manifestation, and transitory course of new or worsened symptoms.^12^

### Study design and clinical assessment battery

Pre-season baseline testing was performed on all study participants. After SRC, athletes were reassessed between 24 and 48 h, and at 8, 15, and 45 days post-injury. A brief assessment was performed at their schools within 6 h of injury (primarily to obtain a blood draw for the parent study). Control athletes completed assessments at equivalent intervals. Relevant components of the baseline assessment consisted of demographic and medical history information and a clinical assessment battery including the following measures: Wechsler Test of Adult Reading (WTAR), Sport Concussion Assessment Tool 3 (SCAT3) symptom checklist, Brief Symptom Inventory-18 (BSI-18), and Pittsburgh Sleep Quality Index (PSQI).

For this sleep-monitoring sub-study, participants were given a commercially available actigraph (CA) and a mobile application developed for the purpose of this study.^13^ Previous literature has reported high concordance between data recorded by the Fitbit Charge HR and clinical actigraphs in young adults and adolescents.^14,15^ The general methodology for this study and the mobile application development has also been described (focused on physical activity metrics) in a prior publication.^13^ Participants were asked to wear the actigraph as often as possible (day and night) for 2 weeks to provide daily sleep and activity metrics. The mobile survey (MS), delivered nightly, asked about total amount of sleep and total time spent in bed the night before. The MS also delivered questions about activities and (for concussed participants) symptoms and recovery, which are not reported here.

The full enrolled sample provided a median of 6 days (interquartile range [IQR] = 1.25–11.00 days) of CA sleep data. A total of 14 (16.7%) participants provided no valid CA sleep data across the 2-week study period. All participants provided at least 1 valid day of MS data, and an overall median of 11 days (IQR = 7.25–13.00 days) of data. A total of 46/971 (4.7%) CA days were dropped from the analysis due to incomplete/invalid data (i.e., step count <1000 or Fitbit HR activity minutes <1000).

### Commercial actigraphy

Participants were provided with a Fitbit Charge HR (version 1 or 2, which are compatible) using firmware version 122 (Fitbit, 116 San Francisco, CA, USA), and were instructed to wear the CA from their 24–48-h assessment through their day 15 in-person assessment. A wrist-based device was selected over one intended to be worn on the waist because we expected the wrist-based device to yield higher compliance and because of our interest in continuously monitoring the heart rate. Research assistants entered each participant's date of birth, height, weight, handedness, and wear location (i.e., dominant vs. non-dominant wrist, per preference) into the Fitbit mobile application during the initial device setup. Control group athletes were instructed to wear the device as often as possible, including while sleeping and during athletic practices and games if allowed by their coaches and/or athletic trainers. Hours of sleep were collected and sleep efficiency (time asleep/time in bed*100) was computed daily for all subjects. Although more highly validated activity sensors are available, the CA was selected because: 1) it is a relatively affordable and popular activity tracker, and 2) because the device would be given to participants as compensation for participation in the study, it was important to select an attractive consumer product.

Sleep efficiency was calculated in the report from the Fitbit for each participant and was averaged across weeks 1 and 2. Then, weeks 1 and 2 were combined for our correlations. The same calculation was made for the Fitbit-recorded hours asleep. These two metrics from the CA were selected to allow direct comparison to the analogous values collected via the MS.

### Mobile survey

A smartphone application (mHealth mobile health technology questionnaire) was developed to survey participants nightly on their self-reported mental activity, physical activity (adapted from the International Physical Activity Questionnaire), stage of recovery, concussion symptoms, and sleep in the previous 24-h period. The MS was installed at each participant's 24–48-h assessment, in which they were instructed to complete the survey each night based on the day's activities and their sleep the previous night. Participants received a notification at 8 PM reminding them to complete the survey and to charge their CA. Upon completion, the data were automatically uploaded and participants were not able to complete another survey until the next day.

Due to changes in the wording regarding sleep-specific questions after the first season of data collection, researchers split the MS sample by season 1 and season 2. In season 1 of data collection, the questions were: (a) “What time did you go to bed?”; (b) “How long did it take you to fall asleep?”; (c) “What time did you get up in the morning?”; and (d) “How many hours of actual sleep did you get?” Participants were told to report their answers in a 24-h format; however, many participants appeared to report using a 12-h format instead, yielding data that appeared to have unresolvable discrepancies. To improve the quality of self-reported data in season 2, participants were asked (a) “How much time did you spend in bed?” and (b) “How many hours of actual sleep did you get?” These questions provided more precise measurements of sleep efficiency, which were calculated in the same manner as was Fitbit sleep efficiency. For the most accurate measurements, only season 2 sleep efficiency data were used from the MS.

### Statistical analysis

Descriptive statistics (i.e., means [M], standard deviations [SD], percentages) and inferential statistics (i.e., independent samples *t* tests, χ^2^ tests) were computed to compare SRC and contact control groups in demographic and pre-season baseline variables. Time spent wearing the CA each day was estimated as the sum of time spent in each of the heart rate-based physical activity intensity zones to enable comparisons between groups in estimated compliance wearing the CA.

Independent samples *t* tests, and effect sizes (Cohen's *d*) were computed to compare CA wear time and primary sleep metrics between groups. The four primary sleep metrics were: MS hours asleep, CA hours asleep, MS sleep efficiency, and CA sleep efficiency. Degrees of freedom were adjusted in cases in which Levene's test suggested heteroscedasticity. MS and CA data were grouped into multi-day bins to provide more stable sleep estimates and reduce the number of statistical comparisons needed. In particular, the SRC group's data were aggregated for days 0–3, 4–7, 8–11, and 12–15. The control group's data were aggregated for the entire 2-week follow-up period to obtain a maximally stable estimate of normal sleep for this subject population, given the relatively small sample size of the control group and a lack of expectation or evidence of systematic changes in the control group's sleep over time. Sensitivity analyses were performed of the main analyses using the same 4-day bins across groups, which yielded no change in the conclusions.

As described in the Results section, we also employed a secondary analysis comparing SRC and control groups on the degree of intrasubject variability (termed intrasubject standard deviations; ISD) across days 0–3 on the four sleep metrics using independent samples *t* tests.^16^

Finally, we examined the degree of association between self-reported (MS) and objective (CA) sleep using Pearson correlations of hours of sleep and sleep efficiency, stratified by group. Similarly, we compared the mean differences between MS and CA sleep, stratified by group, using paired-samples *t* tests. To simplify these analyses, we averaged each of the four sleep metrics across the 2-week data collection period. Statistical analyses were conducted in IBM SPSS Statistics version 24.0 (IBM Corp., Armonk, NY, USA).

## Results

### Sample characteristics and group matching

Table 1 summarizes demographic and pre-season baseline characteristics of the SRC and control groups. In summary, the sample primarily comprised collegiate-level football players (87.7%) who reported minimal baseline psychiatric, concussion, or sleep symptoms. The groups were not statistically significantly different in age, education level, estimated verbal intellectual ability (word reading performance), proportion of athletes with a neurodevelopmental disorder, or baseline symptoms. Estimated overall mean time spent wearing the CA per day was 15.8 h (SD = 4.5) and was not significantly different between groups (*p* = 0.833).

**Table 1.** Sample Characteristics

	SRC (*n* = 57)	Controls (*n* = 26)	
	M (SD) or* n *(%)		*P*
Age (years)	18.00 (1.44)	18.54 (2.12)	0.247
College (vs. high school)	50 (87.7%)	22 (84.6%)	0.699
WTAR standard score	97.58 (14.88)	97.77 (11.41)	0.954
ADHD	6 (10.5%)	3 (11.5%)	0.891
Learning disability	2 (3.5%)	0 (0.0%)	0.334
Sleep disorder	1 (1.8%)	0 (0.0%)	0.493
Prior history of concussion			0.027
0	28 (49.1%)	21 (80.8%)	
1	18 (31.6%)	2 (7.7%)	
2	8 (14.0%)	1 (3.8%)	
3+	3 (5.3%)	2 (7.7%)	
BL hours of sleep (past month)	7.40 (1.12)	7.47 (1.08)	0.832
Rx medication for symptoms	3 (5.3%)		
OTC medication for symptoms	30 (52.6%)		
Alcohol use			
Since injury	14 (24.6%)		
BL, past month	14 (24.6%)	9 (34.6%)	0.343
Illicit drug use			
Since injury	3 (5.3%)		
BL, past month	2 (3.5%)	2 (7.7%)	0.385
BL SCAT symptom severity	2.54 (4.71)	1.24 (2.26)	0.192
BL BSI-18 GSI	43.86 (7.55)	41.54 (5.76)	0.168
BL PSQI total	3.90 (2.05)	3.52 (1.50)	0.501
BL PSQI sleep efficiency	87.47 (12.68)	90.81 (13.81)	0.434
Fitbit wear time (h/day)	15.76 (4.48)	15.51 (5.49)	0.833

Sample size with baseline PSQI data was 41 (SRC group) and 17 (control group).

ADHD, attention deficit and hyperactivity disorder; BL, pre-season baseline; BSI-18 GSI, 18-item Brief Symptom Inventory Global Severity Index T score; M, mean; OTC, over the counter; PSQI, Pittsburgh Sleep Quality Index; Rx, prescription; SCAT, Sport Concussion Assessment Tool version 2/3/5; SD, standard deviation; SRC, sport-related concussion; WTAR, Wechsler Test of Adult Reading.

### Group differences in sleep duration and efficiency

**FIG. 1.**
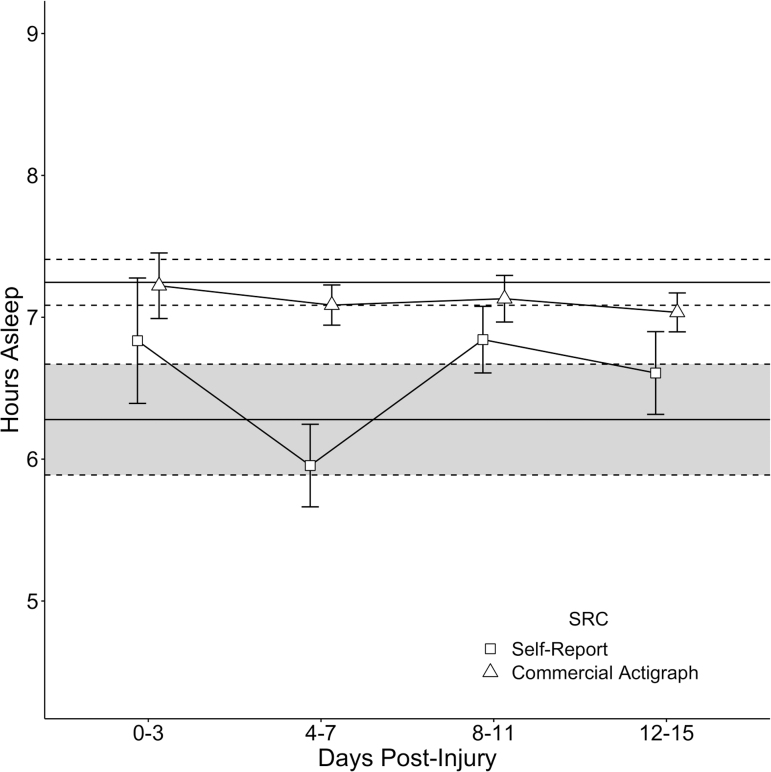
Comparison of SRC and control groups on sleep duration (hours) recorded via CA (triangles) and self-report via mobile application (squares). The SRC group's binned means are presented as shape-dots with ±1 SE error-bars, overlaid onto the 2-week mean of the control group depicted as a solid black line (±1 SE depicted as a continuous band between dotted lines). CA, commercially available actigraphy; SE, standard error; SRC, sport-related concussion.

**FIG. 2.**
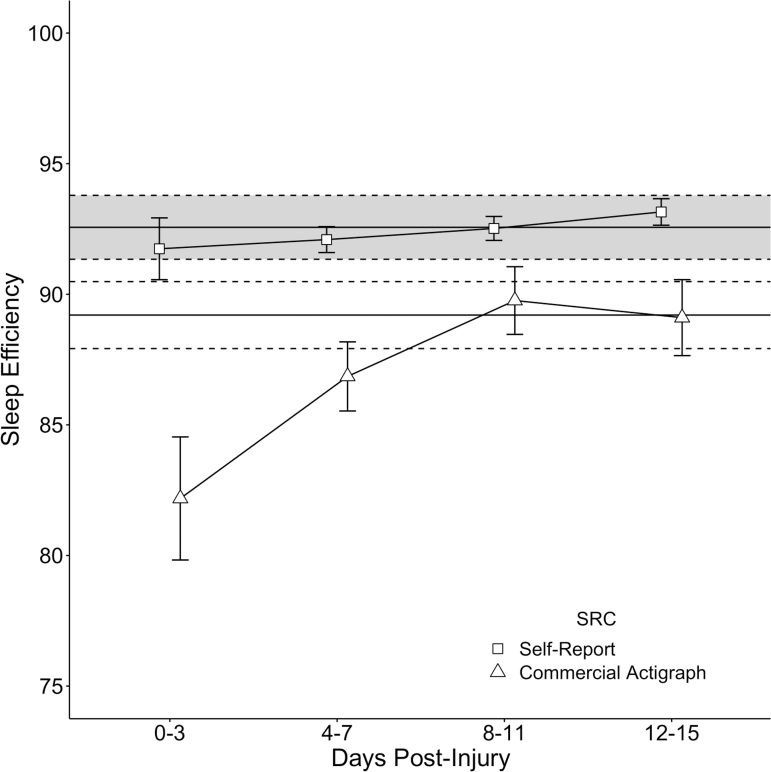
Comparison of SRC and control groups on sleep efficiency (%) recorded via CA (triangles) and self-report via mobile application (squares). The SRC group's binned means are presented as shape-dots with ±1 SE error-bars, overlaid onto the 2-week mean of the control group depicted as a solid black line (±1 SE depicted as a continuous band between dotted lines). CA, commercially available actigraphy; SE, standard error; SRC, sport-related concussion.

Descriptive statistics for the four primary sleep metrics by group are provided in Table 2. Detailed day-by-day descriptive statistics and group comparisons between SRC versus control group metrics are found in Supplementary Table S1. Figure 1 (hours of sleep per CA and MS) and Figure 2 (sleep efficiency % per CA and MS) depict the differences between SRC (binned means = dots, bars = ± 1 standard error [SE]) with and control group (single mean = solid line, band between dotted line = ± 1 SE) on the four primary sleep metrics. As depicted in Figure 1, there were no statistically significant differences between groups on CA-recorded sleep duration or MS-reported sleep duration. As depicted in Figure 2, there were no statistically significant differences between groups on CA-recorded sleep efficiency. However, the SRC group reported lower sleep efficiency on the MS in the first 3 days post-concussion (*d* = −0.77, *p* = 0.013), with this difference resolving by days 4–7 post-injury (*d* = −0.40, *p* = 0.314).

**Table 2.** Descriptive Statistics and Results of Group Comparisons for SRC vs. Control Group Sleep Metrics

	SRC (by day)			Control (2-week average)					
	*N*	M	SD	*N*	M	SD	Levene's *P*	t test *P*	d
Fitbit hours asleep									
Days 0-3				37	6.83	2.69	20	6.28	1.75	**0.033**	0.350	0.25
Days 4–7	44	5.95	1.93							0.190	0.524	0.18
Days 8–11	34	6.84	1.37							0.472	0.194	0.36
Days 12–15	31	6.60	1.62							0.617	0.497	0.19
Fitbit sleep efficiency												
Days 0–3			38	91.74	7.29	20	92.56	5.47	0.569	0.660	0.13	
Days 4–7	44	92.09	3.29						0.557	0.671	0.10	
Days 8–11	34	92.52	2.67						0.375	0.969	0.01	
Days 12–15	31	93.15	2.83						0.456	0.616	0.14	
Self-reported hours asleep												
Days 0–3			50	7.22	1.63	26	7.25	0.82	**0.033**	0.944	0.02	
Days 4–7	54	7.09	1.04						0.306	0.493	0.17	
Days 8–11	54	7.13	1.21						0.351	0.661	0.11	
Days 12–15	48	7.03	0.95						0.303	0.341	0.24	
Self-reported sleep efficiency (season 2 only)												
Days 0–3				27	82.18	12.24	11	89.2	4.25	**0.033**	**0.013**	0.77
Days 4–7	29	86.85	7.13							0.150	0.314	0.40
Days 8–11	29	89.76	6.98							0.058	0.807	0.10
Days 12–15	25	89.10	7.27							0.092	0.967	0.02

Bold: *p* < 0.05.

M, mean; SD, standard deviation; SRC, sport-related concussion.

Because of the minimal relationship identified between CA or MS sleep and acute concussion, we explored the possibility that concussion might be associated with increased variability in sleep duration or efficiency over the acute post-concussive period, as opposed to the mean difference investigated by the aforementioned analyses. Because group differences in MS sleep efficiency were maximal during days 0–3 post-injury, in this secondary analysis, we computed the intrasubject standard deviations (ISD) of each of the four sleep metrics from days 0–3 and submitted this ISD to independent samples *t* tests. This revealed no significant group differences in ISD of CA sleep duration (*p* = 0.918), CA sleep efficiency (*p* = 0.240), MS sleep duration (*p* = 0.964), or MS sleep efficiency (*p* = 0.434).

### Association between objective and self-reported sleep

**FIG. 3.**
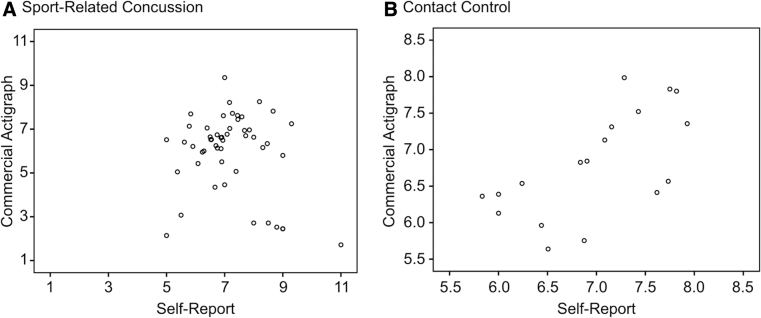
Scatterplot of the relationship between CA-recorded and MS-reported sleep duration (mean number of hours asleep per night across the 2-week study period), stratified by group (**A: **SRC; **B: **contact control). The relationship between self-reported and objective-recorded sleep duration was stronger in the control (r = 0.65) than in the sport-related concussion group (*r* = −0.21). CA, commercially available actigraphy; MS, mobile survey.

Correlational analyses comparing objective (CA) and self-reported (MS) sleep metrics emphasized sleep duration, for which the sample size was largest. For completeness, however, correlations between CA and MS sleep efficiency are also reported. Figure 3 provides a scatterplot of the relationship between CA and MS mean hours of sleep (across the 2-week follow-up period) for the SRC group (Fig. 3A) and the control group (Fig. 3B). The correlation was significant and larger for the control group (*r* = 0.65, *p* = 0.004), versus smaller and non-significant for the SRC group (*r* = −0.21, *p* = 0.145). A similar difference between groups appeared in the correlation magnitudes between CA and MS sleep efficiency (control *r* = 0.66, *p* = 0.226; SRC *r* = 0.05, *p* = 0.798), although sample size precluded the control group correlation (*n* = 5) from being powered for significance.

Paired-samples *t* tests comparing CA with MS sleep revealed no significant differences in mean sleep duration or sleep efficiency between the two modes of assessment for controls: CA sleep duration M = 6.80 (SD = 0.72), MS sleep duration M = 6.97 (SD = 0.68), *p* = 0.237; CA sleep efficiency M = 93.16 (SD = 1.42), MS sleep efficiency M = 89.36 (SD = 5.43), *p* = 0.139. In contrast, participants in the SRC group, on average, self-reported more time asleep (MS sleep duration M = 7.22, SD = 1.21) and poorer sleep efficiency (MS sleep efficiency M = 84.69, SD = 9.55) than was recorded by their CAs (CA sleep duration M = 5.99, SD = 1.79; CA sleep efficiency M = 92.31, SD = 3.55; both *p* < 0.001).

## Discussion

Our prospective pilot study provided preliminary data to: 1) characterize the association between acute-to-subacute SRC and sleep-wake behaviors collected via both objective (CA) and self-reported (MS) methods, and 2) assess the congruence between the two methods of sleep-wake measurement in both subacute SRC and control samples. Regarding the first objective, there was no significant association between SRC and objectively measured sleep duration or efficiency, nor did concussed athletes self-report reduced sleep duration after SRC. The SRC group self-reported lower sleep efficiency compared with the non-injured controls, and this relationship was limited to the first three post-injury. This implies that the subjective experience of sleep disturbance after SRC: 1) may be time-limited to the acute post-injury period, and 2) does not align with comparable objective metrics recorded from a CA, which shows no relationship with concussion.

We observed a strong correlation between self-reported and actigraph-assessed sleep metrics for non-injured controls, supporting the validity of these self-reported sleep metrics among non-injured athletes. In contrast, there was a weak and insignificant relationship between objective and subjective sleep measurements for the SRC group. Notably, the weak correlation strength (*r* ∼ 0.20) between sleep methods within the post-SRC sample was on par with previous work,^17^ although in our study the relationship fell below *a priori* significance threshold. Overall, this implies a disconnect between subjective experience and objective observation of sleep-state following SRC and renders the meaning of the brief reduction in self-reported sleep efficiency after concussion unclear.

Several interpretations could be considered, which, notably, may not be mutually exclusive. First, the sleep loss/low sleep efficiency reported by post-SRC athletes may not reflect changes in actual sleep-wake behaviors. Relatedly, the authors recently demonstrated that post-concussion syndrome symptoms as assessed by the SCAT are essentially unidimensional in that there is a strong correspondence among ratings of diverse concussion symptoms in the acute post-injury period with a single underlying dimension accounting for 96% of variance in concussion symptoms.^18^ This implies that self-reported symptoms may be non-specific in nature, and that something other than sleep, perhaps overall symptom severity or general distress, specifically influences symptom ratings on self-report instruments more than specific concussion-related complaints. This interpretation is supported by findings that subjective sleep disturbance correlates with reported multiple psychosocial distress factors, but not objective indicators of sleep disturbance (per actigraph or polysomnogram).^19,20^ However, other factor analytic studies have found a unique sleep-related symptom dimension^12^ that is present both pre- and post-concussion.^21^ Thus, future work is necessary to clarify these apparently discrepant findings.

Alternatively, it may be that subjective sleep symptoms following SRC are associated with physiological sleep changes not captured by actigraphy-measured sleep-wake behavior. Were this interpretation to be accurate, a behaviorally unapparent alteration in sleep physiology post-injury may mimic the findings in experimental sleep restriction in otherwise healthy individuals (including young adults similar to student-athletes), which has been demonstrated to elevate post-concussion syndrome (PCS) symptoms and impairments in visual memory, motor speed, and reaction time (cognitive deficits common to SRC).^22^ One related consideration is that diurnal sleepiness rather than nocturnal sleep disturbance may be a more notable clinical manifestation of arousal-sleep disturbance following SRC (i.e., sleepiness does not necessarily result in increased sleep behavior if the drive is resisted or if environmental demands prevent sleep), which was not fully explored by the methods of this study.

These results should be interpreted with caution, given study limitations. Considering imperfect CA wear times, we cannot assume that the actigraph data collected completely represented sleep-wake time. However, that we found comparable wear time across groups supports our inferences about concussion-related effects. Similarly, that there were no mean differences between subjective and objective sleep metrics for the control group implies that these participants wore their devices sufficiently to yield valid estimates. Additionally, although actigraph-based measurement of sleep has the advantage of objectivity, it measures sleep-wake behavior rather than the underlying physiological sleep-wake state and sleep architecture (i.e., stages N1, N2, N3, rapid eye movement [REM]). Furthermore, due to the study being a pilot, methodological factors resulted in especially low sample size with data on one sleep measure (self-reported sleep efficiency), which unfortunately was the measure that showed most promise of a concussion-related effect.

Our findings may not generalize outside the primarily male secondary/collegiate football athlete sample studied here. Specifically, the findings may not be representative of the broader mTBI population, for whom acute injury characteristics (e.g., rates of unconsciousness and peri-traumatic amnesia) would suggest a greater range of severity than that seen in athlete populations. Additionally, this study enrolled participants at 24–48 h post-injury, precluding estimates of sleep in the first night post-injury, when sleep disturbance may be most prominent. Finally, the cross-sectional design precluded estimation of pre- to post-injury changes in sleep behavior, which may be more powerful than the group comparisons employed here.

## Conclusion

Prior work suggests that SWD is a common complaint following SRC, yet studies have primarily assessed SWD through self-report inventories that rely on retrospective recall of sleep dysfunction over days or weeks. We prospectively assessed sleep-wake functioning in participants 48 h to 2 weeks post-SRC using two modes of assessment: a mobile application that distributed daily surveys of sleep-wake-related symptoms, and an objective CA. Our findings suggest increased reporting of sleep inefficiency (i.e., less sleep during periods of intended sleep) in the first few days post-injury without concurrent change noted in objective measurement of sleep efficiency. This suggests the possibility that sleep symptoms following concussion do not reflect changes in actual sleep behaviors.

This null result for objectively measured sleep behaviors requires integration with the broader literature supporting post-mTBI SWD as a risk factor of neuropathophysiological changes associated with neurodegenerative conditions (e.g., glymphatic functioning disturbance), along with exacerbation of post-concussion syndrome-related factors including somatic complaints (e.g., pain, fatigue, headache), psychiatric conditions (e.g., post-traumatic stress disorder, depression, anxiety), cognitive function, and health-related quality of life.^9^ Given this body of work, we continue to believe that clinical research investigating whether SWD exacerbates acute post-concussion symptomatology and/or contributes to the manifestation of self-sustaining/persisting chronic concussion symptoms is warranted.^23^

Our findings suggest that such future work should incorporate physiological measurement of sleep-wake functions after SRC to determine whether symptoms are associated with other underlying disturbances to sleep-related neurological processes associated with quality of sleep, but not behavioral changes in sleep-wake functions. Additionally, capturing intra-individual pre-injury sleep functioning baseline would add power to future studies. Further, *a priori* methods and planned analyses should consider accounting for proposed moderating factors of SWD following SRC (and mTBI more broadly), including trauma characteristics (e.g., strain in the axonal direction, degree of brainstem distortion, functional network disruption in the insula and cingulate cortex), genetics (e.g., PER3 polymorphism), anthropomorphic features (e.g., shallow tentorial angle), and sociodemographic characteristics (e.g., older age, female gender).^24^

## Supplementary Material

Supplemental data

## Abbreviations Used

BSI-18: Brief Symptom Inventory
CA: commercially available actigraphy
IQR: interquartile range
ISD: intrasubject standard deviations
M: mean
MS: mobile survey
mTBI: mild traumatic brain injury
PSQI: Pittsburgh Sleep Quality Index
REM: rapid eye movement
SCAT3: Sport Concussion Assessment Tool 3
SD: standard deviation
SE: standard error
SRC: sport-related concussion
SWD: sleep-wake disturbance
WTAR: Wechsler Test of Adult Reading
