# Supplementary material for: Relationship between Sport-Related Concussion and Sleep Based on Self-Report and Commercial Actigraph Measurement
Source: Neurotrauma Rep. 2021 Apr 26;2(1):214–23. doi: 10.1089/neur.2021.0008 (PMC8086521; doi:10.1089/neur.2021.0008)
Supplement: Supplemental data [file Supp_Table1.docx]

**Supplementary Table**. Descriptive statistics and results of group comparisons for SRC vs. control group sleep metrics.

|  | **SRC (by day)** | | | **Control (2-week average)** | | | | |  |
| --- | --- | --- | --- | --- | --- | --- | --- | --- | --- |
| Fitbit hours asleep | N | M | SD | N | M | SD | Levene's p | t-test  p | d |
| D1 | 6 | 6.57 | 5.59 | 20 | 6.2789 | 1.74776 | .030 | .906 | .07 |
| D2 | 20 | 5.98 | 2.79 |  |  |  | .020 | .692 | -.13 |
| D3 | 35 | 6.93 | 2.82 |  |  |  | .035 | .294 | .28 |
| D4 | 32 | 6.09 | 2.15 |  |  |  | .194 | .746 | -.10 |
| D5 | 30 | 6.27 | 2.20 |  |  |  | .073 | .983 | -.01 |
| D6 | 28 | 5.75 | 1.98 |  |  |  | .359 | .345 | -.28 |
| D7 | 29 | 6.31 | 1.92 |  |  |  | .338 | .949 | .02 |
| D8 | 26 | 7.06 | 1.52 |  |  |  | .793 | .115 | .47 |
| D9 | 24 | 6.93 | 1.64 |  |  |  | .921 | .214 | .38 |
| D10 | 25 | 6.98 | 1.89 |  |  |  | .320 | .205 | .39 |
| D11 | 24 | 6.08 | 1.88 |  |  |  | .380 | .717 | -.11 |
| D12 | 23 | 6.56 | 2.16 |  |  |  | .388 | .640 | .15 |
| D13 | 20 | 7.05 | 1.43 |  |  |  | .825 | .134 | .48 |
| D14 | 22 | 6.34 | 2.19 |  |  |  | .076 | .924 | .03 |
| D15 | 16 | 5.87 | 1.39 |  |  |  | .967 | .451 | -.26 |
| Fitbit sleep efficiency |  |  |  |  |  |  |  |  |  |
| D1 | 6 | 93.67 | 5.72 | 20 | 92.5624 | 5.47048 | .518 | .671 | .20 |
| D2 | 20 | 92.55 | 4.45 |  |  |  | .543 | .994 | .00 |
| D3 | 35 | 91.54 | 7.82 |  |  |  | .347 | .609 | -.15 |
| D4 | 32 | 92.09 | 3.27 |  |  |  | .628 | .700 | -.10 |
| D5 | 30 | 92.00 | 3.95 |  |  |  | .804 | .675 | -.12 |
| D6 | 28 | 92.29 | 3.41 |  |  |  | .836 | .830 | -.06 |
| D7 | 29 | 91.28 | 4.89 |  |  |  | .771 | .393 | -.25 |
| D8 | 26 | 92.23 | 3.65 |  |  |  | .991 | .807 | -.07 |
| D9 | 24 | 93.42 | 2.83 |  |  |  | .635 | .509 | .20 |
| D10 | 25 | 92.32 | 3.82 |  |  |  | .708 | .862 | -.05 |
| D11 | 24 | 92.71 | 3.03 |  |  |  | .673 | .911 | .03 |
| D12 | 23 | 92.22 | 3.10 |  |  |  | .748 | .797 | -.08 |
| D13 | 20 | 92.85 | 3.75 |  |  |  | .995 | .847 | .06 |
| D14 | 22 | 92.86 | 3.58 |  |  |  | .999 | .832 | .07 |
| D15 | 16 | 93.50 | 2.85 |  |  |  | .569 | .539 | .21 |
| Self-reported hours asleep |  |  |  |  |  |  |  |  |  |
| D1 | 21 | 6.93 | 1.78 | 26 | 7.2463 | 0.82395 | .003 | .453 | -.23 |
| D2 | 35 | 7.23 | 2.07 |  |  |  | .001 | .968 | -.01 |
| D3 | 46 | 7.36 | 1.73 |  |  |  | .012 | .695 | .09 |
| D4 | 46 | 7.14 | 1.36 |  |  |  | .012 | .685 | -.09 |
| D5 | 46 | 7.21 | 1.30 |  |  |  | .058 | .887 | -.04 |
| D6 | 45 | 7.23 | 1.51 |  |  |  | .010 | .939 | -.02 |
| D7 | 46 | 7.02 | 1.69 |  |  |  | .020 | .443 | -.17 |
| D8 | 43 | 7.43 | 1.59 |  |  |  | .019 | .535 | .14 |
| D9 | 44 | 7.24 | 1.47 |  |  |  | .035 | .974 | -.01 |
| D10 | 38 | 7.07 | 1.36 |  |  |  | -- | -- |  |
| D11 | 38 | 7.00 | 1.21 |  |  |  | .246 | .373 | -.24 |
| D12 | 41 | 6.98 | 1.34 |  |  |  | .055 | .368 | -.24 |
| D13 | 38 | 7.14 | 1.24 |  |  |  | .031 | .693 | -.10 |
| D14 | 33 | 7.60 | 1.38 |  |  |  | .033 | .221 | .32 |
| D15 | 32 | 6.88 | 1.57 |  |  |  | .048 | .253 | -.30 |
| Self-reported sleep efficiency (season 2 only) | | |  |  |  |  |  |  |  |
| D1 | 16 | 79.71 | 17.44 | 11 | 89.2032 | 4.25148 | .001 | *.052* | -.75 |
| D2 | 22 | 82.91 | 14.36 |  |  |  | .015 | *.069* | -.59 |
| D3 | 24 | 82.88 | 12.55 |  |  |  | .010 | **.035** | -.68 |
| D4 | 22 | 84.77 | 13.75 |  |  |  | .029 | .177 | -.44 |
| D5 | 22 | 88.29 | 8.06 |  |  |  | .049 | .672 | -.14 |
| D6 | 24 | 88.41 | 7.60 |  |  |  | .180 | .751 | -.13 |
| D7 | 22 | 86.64 | 11.78 |  |  |  | .057 | .492 | -.29 |
| D8 | 22 | 89.63 | 8.49 |  |  |  | .014 | .850 | .06 |
| D9 | 23 | 90.88 | 8.21 |  |  |  | .070 | .529 | .26 |
| D10 | 19 | 88.48 | 11.06 |  |  |  | .001 | .802 | -.09 |
| D11 | 16 | 90.26 | 6.95 |  |  |  | .024 | .630 | .18 |
| D12 | 20 | 89.57 | 8.74 |  |  |  | .143 | .897 | .05 |
| D13 | 17 | 86.04 | 9.90 |  |  |  | .066 | .328 | -.42 |
| D14 | 15 | 87.84 | 8.50 |  |  |  | .066 | .629 | -.20 |
| D15 | 13 | 92.38 | 6.59 |  |  |  | .002 | .170 | .57 |

*Note*. D#, day; SRC, sport-related concussion group; **p < 0.05**; *p < 0.10*
